# Malignant Hypertension Causing a Pulmonary-Renal Syndrome

**DOI:** 10.1155/2018/3273695

**Published:** 2018-12-17

**Authors:** Bryan Yong, David A. Power

**Affiliations:** ^1^Clinical School, University of Melbourne, Parkville, Victoria, Australia; ^2^Department of Nephrology, Austin Health, Studley Road, Heidelberg 3084, Victoria, Australia

## Abstract

**Background:**

Pulmonary-renal syndrome is characterised by acute kidney injury, haematuria, and haemoptysis and is a well-recognised presentation of diseases such as ANCA vasculitis that require urgent immunosuppression.

**Case Presentation:**

A patient presented with a brief history of haemoptysis, acute renal failure, microscopic haematuria, and severe hypertension. The diagnosis was initially not clear so he was treated with antihypertensives, renal replacement therapy, and immunosuppression. Renal biopsy subsequently showed evidence of malignant hypertension. Autoantibodies were uniformly negative.

**Conclusions:**

This case demonstrates that malignant hypertension can present as pulmonary-renal syndrome.

## 1. Background

Pulmonary haemorrhage with acute kidney injury and haematuria is classically caused by immunologically mediated diseases such as ANCA-associated vasculitis, anti-GBM disease, which is also known as Goodpasture's syndrome, or systemic lupus erythematosus [1]. In the acute clinical setting, it is often necessary to treat patients with immunosuppressive agents and plasma exchange prior to accurate diagnosis. We present an unusual case of haemoptysis and acute kidney injury, treated with initial immunosuppression, where hypertension was found to be the causative problem.

## 2. Case Summary

A thirty-three-year-old Indian gentleman presented with a two-week history of haemoptysis and a three-day history of fever, epigastric discomfort, nausea, and headache. This occurred on a background of hypertension, diagnosed 8 years previously. He was a smoker with a 5 pack-year smoking history. He took no regular or over the counter medications.

**Figure 1:**
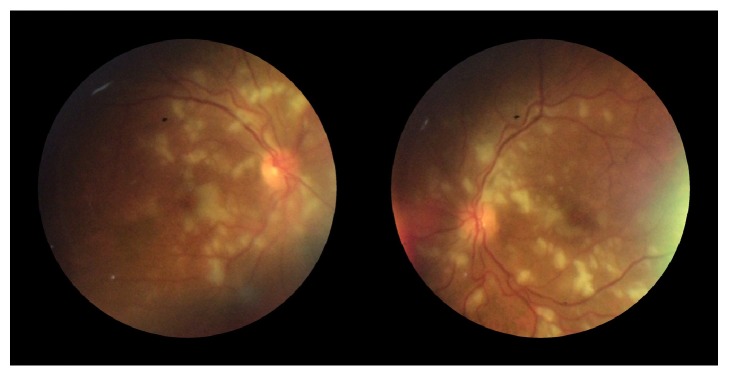
Right and left funduscopic images showing flame haemorrhages and cotton wool spots, consistent with grade 3 hypertensive retinopathy.

At presentation, he was markedly hypertensive (225/145 mmHg) and tachycardic (110 bpm). He was afebrile with normal oxygen saturations and had dual heart sounds with a loud P2. Clinical examination indicated euvolemic fluid status. There were bilateral mid and lower zone inspiratory crackles and he had small volume haemoptysis. There were no focal neurological signs. Optic fundoscopy revealed flame haemorrhages and cotton wool spots, consistent with grade 3 hypertensive retinopathy (Figure 1). An electrocardiogram revealed high amplitude QRS complexes in V1 and V6 consistent with left ventricular hypertrophy. The echocardiogram showed normal left ventricular size but moderate to severe left ventricular wall thickness.

On admission, he had a normocytic anaemia (Hb 75 g/L, RR = 130-180 g/L) and thrombocytopenia (platelets 98 × 10^9^/L, RR = 150-400 × 10^9^/L). The serum creatinine was elevated at 810 *μ*mol/L (RR = 62-106 *μ*mol/L), with an estimated glomerular filtration rate (eGFR) of 7 mL/min (RR = ≥90 mL/min) (Table 1). The haemolysis screen revealed a bilirubin of 46 *μ*mol/L (RR = <18 *μ*mol/L), reticulocyte count of 333 × 10^9^/L (RR = 20-100 × 10^9^/L), lactate dehydrogenase (LDH) of 1177 IU/L (RR = 135-225 IU/L), and a haptoglobin of <0.1 mg/L (RR = 0.3-2.0 mg/L). The peripheral blood smear showed moderate schistocytosis and moderate polychromasia, in keeping with haemolysis (Table 1). A dipstick was positive for proteinuria (2+) and haematuria (2+). Urine microscopy showed 40 × 10^6^ erythrocytes (RR < 40 × 10^6^). Ultrasound revealed normal sized kidneys without hydronephrosis. Corticomedullary differentiation was normal. The chest X-ray showed cardiomegaly, patchy bilateral airspace opacities that were worse on the right side.

The clinical suspicion was that of an ANCA vasculitis or anti-GBM disease, with a differential diagnosis that included complement mediated haemolytic uraemic syndrome (HUS), thrombotic thrombocytopaenic purpura (TTP), and malignant hypertension.

**Figure 2:**
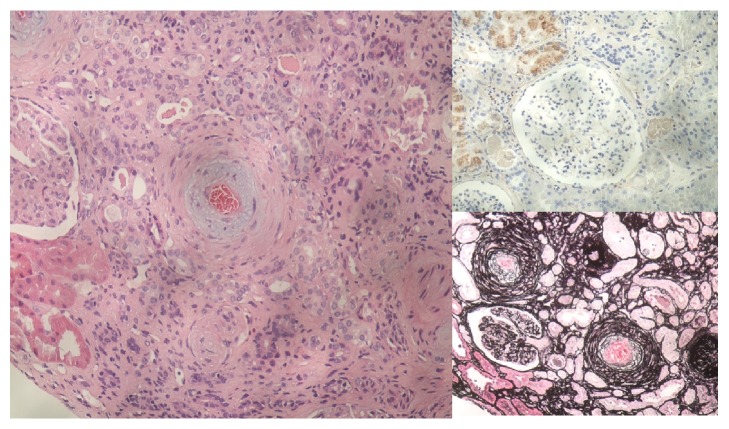
Renal biopsy. Left: arteriolar thickening and myxoid changes. Top right: negative immunofluorescence staining. Bottom right: onion skinning of arterioles on silver staining.

The hypertension was treated with metoprolol, methyldopa, and hydralazine to achieve a BP of 150/100. Pulse methylprednisolone therapy, followed by cyclophosphamide, and plasma exchange were commenced. The headache, haemoptysis, and evidence of haemolysis rapidly resolved following this treatment. Renal biopsy was initially delayed due to hypertension and was subsequently performed on day 3 (Figure 2). The biopsy contained 18 glomeruli. Most glomeruli showed a consolidated, bloodless appearance with obscuration of capillary loops by endothelial cell swelling and subendothelial zone expansion. Staining for IgA, IgM, IgG, fibrinogen, C3c, and C1q was negative. The biopsy suggested malignant hypertensive nephropathy as the underlying cause of the acute kidney injury. Therapy with steroids, cyclophosphamide, and plasma exchange was ceased once the renal biopsy results became available. Furthermore, the autoimmune screening panel which included antinuclear antibodies (ANA), antinuclear cytoplasmic antibodies (ANCA), anti-GBM antibodies, extractable nuclear antibodies (ENA), and complement levels was normal. The ADAMTS13 levels in addition were later found to be normal and did not suggest complement mediated HUS.

Control of hypertension was associated with resolution of the biochemical evidence of haemolysis. CT angiogram showed no evidence of renal artery stenosis. The patient remained dialysis dependent for 7 months and then regained sufficient renal function to cease dialysis. Fourteen months after presentation, the BP is 127/87 with serum creatinine 337 *μ*mol/L and eGFR 19 ml/min. Current medications are perindopril, indapamide, carvedilol, frusemide, calcitriol, and rosuvastatin. A recent echocardiogram shows left ventricular hypertrophy and low normal systolic function. He remains under regular review by cardiology and nephrology.

## 3. Discussion

Pulmonary haemorrhage with acute kidney injury is classically caused by immunologically mediated diseases such as ANCA associated vasculitis, Goodpasture's syndrome, or systemic lupus erythematosus [1]. The combination of hypertension, hypertensive changes on fundoscopy, laboratory evidence of microangiopathic haemolytic anaemia, and a renal biopsy consistent with thrombotic microangiopathy suggested that the unifying diagnosis in this case was malignant hypertension. Renal biopsy findings and negative ANCA, anti-GBM, and ANA serologies were supportive of a nonimmunological cause. Clinical and biochemical improvements with strict hypertension control also supported the diagnosis of malignant hypertension.

Malignant hypertension typically presents with nonspecific symptoms such as agitation, an altered conscious state, chest discomfort, and headache. It is a rare cause of pulmonary-renal syndrome [2–5]. As we were unable to perform a kidney biopsy due to hypertension, it was difficult to exclude an immunological cause for the pulmonary-renal syndrome. The patient presented on the weekend and it proved impossible to obtain emergency serology, which might have helped to exclude several of the diagnoses entertained. This would likely have spared the patient unnecessary immunosuppression. It is also worth noting that severe hypertension is unusual in patients with anti-GBM disease and ANCA vasculitis, which is described in a few case series [6, 7].

This patient had clear evidence of intravascular haemolysis. There were schistocytes observed in the peripheral blood smear, which would support a diagnosis of atypical HUS. Pulmonary haemorrhage is considered quite unusual in aHUS. The diagnosis was considered possible, however, until the prompt resolution of haemolysis with control of blood pressure suggested that aHUS was unlikely. The appearances of malignant hypertension on renal biopsy are similar to that of aHUS when thrombotic microangiopathy (TMA) is present. In TMA, the appearances on renal biopsy include intravascular and glomerular thrombi as well as mesangial changes. In malignant hypertension, however, more severe vascular changes in small arterioles would be expected.

The mechanism of haemoptysis in malignant hypertension is unclear. Malignant hypertension may cause pulmonary haemorrhage via direct vascular endothelial injury from mechanical stress. Elevated left ventricular pressure and pulmonary oedema causing pulmonary haemorrhage may be an alternate mechanism. It was reported that 63% of patients with malignant hypertension had renal impairment on presentation [8]. Renal survival was notably found to be 84% and 72% at five and ten years, respectively.

While immunological causes of pulmonary-renal syndrome are more common, malignant hypertension should be considered in the differential diagnosis in the correct clinical setting. Hypertension management alone may result in improvement in haemolysis markers and pulmonary symptoms; however long term renal outcome remains poor.

## Figures and Tables

**Table 1:** Laboratory results.

**FBC**	**Hb**	**MCV**	**WCC**	**Platelets**	
	75	87	11.3	98	

*Ref. range*	*130-180 g/L*	*80-96 FL*	*4-11 x 10* ^*9*^ */L*	*150-400 x 10* ^*9*^ */L*	

**UEC**	**Na**	**K**	**Cr**	**eGFR**	

	135	2.5	810	7	

*Ref. range*	*136-145 mmol/L*	*3.5-5.1 mmol/L*	*62-106 µmol/L*	*≥90 mL/min*	

**Haemolysis screen**	**Bilirubin**	**Reticulocytes**	**LDH**	**Haptoglobin**	

	46	333	1177	<0.1	

*Ref. range*	*<18 µmol/L*	*20-100 x 10* ^*9*^ */L *	*135-225 IU/L *	*0.3-2.0 mg/L*	

**Blood smear**	Moderate schistocytosis and polychromasia				

**Serology**	**ANA**	**ANCA**	**Anti-GBM**	**ENA panel**	**ADAMTS13**

	Negative	Negative	<5	Negative	52

*Ref. range*			*<20 U/mL*		*40-130*%
